# Internet gaming disorder, social network disorder and laterality: handedness relates to pathological use of social networks

**DOI:** 10.1007/s00702-014-1361-5

**Published:** 2015-01-10

**Authors:** Polyxeni Bouna-Pyrrou, Christiane Mühle, Johannes Kornhuber, Bernd Lenz

**Affiliations:** Department of Psychiatry and Psychotherapy, Friedrich-Alexander University Erlangen-Nürnberg (FAU), Schwabachanlage 6-10, 91054 Erlangen, Germany

**Keywords:** Internet addiction, Internet gaming disorder, Social network, Laterality, Handedness

## Abstract

The internet age bears new challenges that include health risks. It is agreed that excessive internet use may reach pathological levels. However, the concept of internet addiction lacks specificity and, therefore, warrants studies on its diagnostic and etiologic classification. This study was conducted to characterize the novel DSM-5 criteria for internet gaming disorder and the adapted criteria for the “social network disorder”. Based on the established association of handedness and substance use disorders, we also explored whether internet use related to laterality. For this study, 3,287 volunteers participated in the online survey and gave particulars concerning their internet use in general, internet gaming and use of social networks, laterality markers (hand, foot, eye, ear, rotational preference in gymnastics, and head turning asymmetry) and health status. Of the participants, 1.1 % fulfilled the criteria for internet gaming disorder, and 1.8 % fulfilled the criteria for social network disorder. The applied criteria were highly correlated with the time spent on the respective internet activities (*p* < 4 × 10^−56^). The analyses of comorbidity and working hours support the thresholds of 5/9 criteria and ≥30 h/week spent on the internet for the classification as pathological (*p* < 5 × 10^−2^). Moreover, we found that left-handedness related to more affirmed criteria and longer times spent on social networks (*p* ≤ 4 × 10^−2^). The provided criteria proved to be user-friendly, comprehensible and well accepted. The results contribute to a better understanding of pathological internet gaming and social network use and provide evidence that biological markers of substance use disorders are involved in internet addiction.

**Supplementary Information:**

The online version contains supplementary material available at 10.1007/s00702-014-1361-5.

## Introduction

Due to the widespread availability of the internet, individuals can engage in a growing number of fascinating activities. Epidemiological studies indicate that approximately 1–2 % of the population suffers from internet addiction (Aboujaoude et al. 2006; Bakken et al. 2009; Rumpf et al. 2013; Sussman et al. 2011), but this should be interpreted cautiously because of the lack of approved criteria. Pathological internet use is associated with numerous physical and psychiatric problems, including decreased well-being and social skills (Lemmens et al. 2011), poor job performance (Young 1999), impaired sleep quality (Andreassen et al. 2012; Dworak et al. 2007; Wolniczak et al. 2013), and depressive and anxiety disorders (Carli et al. 2013; Ko et al. 2012; Morrison and Gore 2010; Spada 2014; Weinstein and Lejoyeux 2010).

In May 2013, the American Psychiatric Association (APA) included the internet gaming disorder (IGD) in the 5th edition of the diagnostic and statistical manual of mental disorders (American Psychiatric Association 2013) as a condition warranting more research. Further knowledge on the diagnostic and etiologic classification of IGD and similar potentially addictive activities is needed (Holden 2010; Petry et al. 2014). In particular, excessive use of social network sites has been suggested as another new type of behavioral addiction (Andreassen and Pallesen 2014; Kuss and Griffiths 2011). Griffiths (2005) proposed that addictions share common core components. Different questionnaires for the pathological use of social network sites, which were developed in recent years, have been found to overlap with the novel diagnostic criteria for IGD (Andreassen and Pallesen 2014). Thus, we decided to use the term “social network disorder” (SND; analogous to IGD in DSM-5) and adapted the nine DSM-5 criteria for IGD to the pathological use of social networks with the aim to characterize the concept of SND in comparison to IGD.

In recent years, we used various biomarkers for intrauterine sex hormone activity and found evidence that prenatal testosterone exposure modulates the risk for alcohol use (Kornhuber et al. 2011; Lenz et al. 2012) and video gaming disorders (Kornhuber et al. 2013). These findings motivated us to follow the early sex hormone model of addictive behavior, which assumes that the intrauterine sex hormone priming of the cerebral reward system predisposes individuals to the development of addictive disorders. Cerebral lateralization is also a biomarker for prenatal androgen load (Cohen-Bendahan et al. 2005; Geschwind and Galaburda 1985; Witelson 1985), and its proxy handedness has been repeatedly linked to substance use and addictive behavior. Denny showed that left-handers drink more alcohol than do right-handers (Denny 2011), and many studies consistently demonstrate that alcohol-addicted patients are more often left-handed than are healthy persons (Harburg 1981; London et al. 1985; McNamara et al. 1994; Nasrallah et al. 1983; Sperling et al. 2000, 2010). Moreover, Preti et al. (2012) reported that left-handers experiment more often with heroin, ecstasy and hallucinogens than non-left-handers.

Here, we set two objectives to promote the diagnostic and etiologic characterization of pathological internet use. First, we employed the novel DSM-5 criteria for the IGD and the adapted criteria for SND to evaluate their applicability and their relation to time spent on the specific activities, psychiatric co-morbidities and life style using an online survey. Second, we explored whether these criteria and leisure time use of the internet in general, of internet games and of social networks were related to laterality (handedness, footedness, eyedness, earedness, rotational preference in gymnastics, and head-turning asymmetry).

## Methods

### Study sample

This study was conducted in accordance with the principles expressed in the 6th revision of the Declaration of Helsinki, Seoul 2008 and was approved by the Ethics Committee of the Friedrich-Alexander University Erlangen-Nürnberg (FAU). Interested parties were motivated to participate by the chance to win Amazon gift cards in a prize draw. Between October 2013 and March 2014, 3,287 volunteers started the 20-min standardized online survey published on the online platform SoSci Survey (www.soscisurvey.de). Of these, 1,441 participants were recruited from the SoSci Panel (Leiner 2014), and 1,846 were attracted through e-mail, social networks and public postings. The participants were excluded from analysis if they stated that they earn their living with internet gaming or were shown to have answered dishonestly, or if the data set failed to meet the SoSci quality score “DEGRADE” of at most 200 as a combined normalized indicator for missing answers and too rapid completion. Altogether, 2,595 data sets included the criteria for IGD, and 2,565 data sets the SND criteria, of which at least 2,506 covered the time spent on the respective activities.

### Measurements

#### Health status

The participants were asked about their subjective health status on a 5-point scale (excellent, very good, good, moderate, poor), their number of medical and psychological consultations during the previous 12 months due to somatic or psychiatric disorders, and the amount of time that they had spent on sports activity and sleeping. Alcohol use was assessed using the CAGE test (Ewing 1984). Regarding smoking behavior, we divided the participants into smokers, non-smokers and ex-smokers.

#### Internet use

Use of the internet in general was assessed by the Compulsive Internet Use Scale [CIUS, (Meerkerk et al. 2009), German version (Wartberg et al. 2014)], which offers a valid measurement of internet use and strongly correlates with the weekly duration of private internet use (Gürtler et al. 2014). Regarding IGD, we used the novel DSM-5 criteria (American Psychiatric Association 2013; Rehbein et al. 2013) to create a German questionnaire (Table 2). Affirmative answers to the nine dichotomous items (yes/no) were summed to a total score. Analogous to the nine proposed criteria for IGD, we designed a German questionnaire for the pathological use of social networks (Table 2), which was defined as chatting and mailing via social networks (e.g., Facebook, twitter, WhatsApp, Hangouts and other messenger services). The participants stated their average time spent on internet games or social networks during the previous 12 months. Then, they were asked to bring the two weeks during which they had used the internet the most during the preceding year to mind and report their maximum time spent on internet gaming or social networks.

#### Laterality

We applied the Waterloo Handedness Questionnaire-Revised (WHQ-R, 36 items) and the Waterloo Footedness Questionnaire-Revised (WFQ-R, 18 items) (Elias et al. 1998) translated into German. Eye and ear preference were measured by three and four questions from the lateral preference inventory (Coren 1993) in a German-modified version (Büsch 2009). We omitted the question concerning the preferred eye to look through the eyepiece of a rifle because it depends on handedness. In addition, ocular dominance was assessed by three repetitions of the Miles test (Miles 1928). Two elements of rotational preference in gymnastics were asked: the direction of rotation in a straight jump with a half turn and the preferred hand placed on the ground to start a cartwheel (Heinen 2012). To determine the head-turning asymmetry, we asked the participants which side they turned their head to when kissing a person on the lips (Güntürkün 2003). For all markers, we used 5-point scale answers that ranged from −2 to 2 (always left, usually left, equal, usually right and, always right; see Online Resource for the German questionnaires).

### Data processing and statistical analysis

#### *Characterization of the DSM*-*5 criteria for IGD and the adapted SND criteria*

Group comparisons were performed using the Mann–Whitney *U* and Kruskal–Wallis tests. We applied two-sided Spearman’s tests to evaluate the basal bivariate correlations and *χ* ^2^ tests for differences in the frequencies. The two-tailed Fisher’s exact test was used if at least one cell failed to reach an expected value of five observations. To confirm the findings, we used multivariate (MANCOVA) and univariate analysis of covariance (ANCOVA), and these were corrected for potential confounding factors [sex, smoking status, educational status (fixed factors), age, drinking status, and body mass index (BMI) (covariates)]. The Kuder-Richardson Formula 20 (KR-20) coefficient was calculated to estimate the internal consistency reliability. Receiver Operating Characteristic (ROC) and Youden’s J statistic (*J* = sensitivity + specificity − 1) were applied to evaluate the thresholds of the time spent on the respective internet activities to discriminate the affected from the healthy individuals (Akobeng 2007).

#### Laterality and pathological internet use

In the exploratory step, we analyzed the effects of all laterality markers on the CIUS score, the number of affirmed IGD and SND criteria and the average and maximum time spent on the respective leisure time activities in a discovery subsample of 790 individuals who had given full particulars (MANCOVA including confounding factors). Afterward, we tested whether the initial finding could also be found in an increased cohort of 2,330 individuals. Finally, we investigated whether handedness also predicted that the participants used social networks ≥30 h/week (ANCOVA). *P* < 5 × 10^−2^ was considered statistically significant. The continuous variables with a significant deviation from the normal distribution (Kolmogorov–Smirnov test) were transformed into ranks (skewness and excess kurtosis between −1.6 and 1.9) prior to their use in parametric statistics (Conover and Iman 1981). The data were analyzed using IBM SPSS statistics Version 21 for Windows (SPSS Inc., Chicago, IL, USA) and Graph Pad Prism 5 (Graph Pad Software Inc., San Diego, CA, USA).

## Results

### Characterization of the DSM-5 criteria for IGD and the adapted SND criteria

The male and female participants differed significantly in several demographic parameters (Table 1) and were, therefore, analyzed independently whenever indicated.

**Table 1:** Demographic characterization

	♀ (*n* = 1,524)	♂ (*n* = 941)	Data sets (%)	*p* value
Age (years)^b^	27 (23/39)^a^	30 (24/43)^a^	100 %	**5** **×** **10** ^**−5**^
Time spent on paid work (hours/week)	16 (0/39)^a^	20 (0/40)^a^	67 %	**8** **×** **10** ^**−4**^
Body weight (kg)	64 (58/74)^a^	80 (72/90)^a^	97 %	**<10** ^**−99**^
Body height (cm)	168 (164/172)^a^	180 (176/185)^a^	97 %	**<10** ^**−99**^
BMI (kg/m^2^)	22.6 (20.5/26.0)^a^	24.3 (22.1/27.5)^a^	97 %	**6** **×** **10** ^**−22**^
Civil status				
Living in a partnership	65.0 %	57.8 %	97 %	**4** **×** **10** ^**−4**^
Married	23.9 %	27.3 %	92 %	7 × 10^−2^
Divorced	7.0 %	6.0 %	86 %	4 × 10^−1^
Level of education			97 %	**5** **×** **10** ^**−3**^
No graduation	3.6 %	2.3 %		
Lower secondary schooling	0.3 %	1.4 %		
Secondary schooling	12.9 %	11.7 %		
Higher educational level	83.2 %	84.6 %		
Smoking status			99 %	
Smoker	14.3 %	16.8 %		**1** **×** **10** ^**−2**^
Non-smoker	71.5 %	65.8 %		
Ex-smoker	14.3 %	17.3 %		

The *p*-values show the results from the Mann–Whitney *U* and *χ* ^2^ tests

*BMI* body mass index

^a^Median, 25/75 %; *p* < 5 × 10^−2^ in bold print

^b^Obligatory answer

The participants reported no major problems in comprehending and answering the DSM-5 criteria for IGD or the adapted SND criteria. The KR-20 coefficients associated with the DSM-5 criteria for IGD were .716 (♀ .669, ♂ .743), and those associated with the SND criteria were .666 (♀ .678, ♂ .631). For a better understanding of the two concepts, we investigated the IGD- and SND-specific patterns of affirmed criteria in our cohort. The female participants affirmed all of the SND items more often than the male participants did, and the converse was true for all of the IGD items. Moreover, the women affirmed each SND criteria significantly more often than the equivalent IGD criteria. This pattern was inconsistent in the males. With regard to single items, we found further interesting similarities and differences between IGD, SND, females and males. Independently for the whole cohort as well as for the female and male subsample, IGD and SND have in common that “loss of control” was among the two most frequently affirmed criteria; whereas “jeopardy or loss of a significant relationship” was among the two least often affirmed criteria. To identify gender dimorphisms, we analyzed sex differences (Δ_♀ − ♂_) regarding the percentage of affirmed IGD and SND criteria (Table 2). The three greatest differences for IGD were that males affirmed “continued use despite adverse consequences” (Δ_♀ − ♂=_−4.8 %), “tolerance” (−3.3 %) and “use of internet to escape or relieve a negative mood” (−3.2 %) more often than females and for SND that females affirmed “use of internet to escape or relieve a negative mood” (6.5 %), “tolerance” (3.8 %) and “loss of control” (3.2 %) more often than males. Finally, we calculated the differences of the aforementioned gender dimorphisms between IGD and SND [=IGD(Δ_♀ − ♂_) – SND(Δ_♀ − ♂_)] and found the three greatest differences for “use of internet to escape or relieve a negative mood” (−9.7 %), “tolerance” (−7.1 %) and “continued use despite adverse consequences” (−6.3 %). This illustrates that those criteria are predominantly related to social network sites in females and to internet games in males (Table 2).

**Table 2:** Frequency of affirmed IGD and SND criteria for the whole sample (“WS”) and divided by sex

	Percent of affirmed criteria						*p* values				
	IGD (%)			SND (%)			♀ vs. ♂		IGD vs. SND		
	WS	♀	♂	WS	♀	♂	IGD	SND	WS	♀	♂
Preoccupation	2.5	1.5	4.0	11.7	12.7	10.2	**9** **×** **10** ^**−5**^	6 **×** 10^−2^	**6** **×** **10** ^**−3**^	**2** **×** **10** ^**−2**^	1 × 10^−1^
Withdrawal symptoms	0.8	0.7	0.9	5.0	6.0	3.3	7 **×** 10^−1^	**2** **×** **10** ^**−3**^	**2** **×** **10** ^**−4**^	**2** **×** **10** ^**−4**^	2 × 10^−1^
Tolerance	4.7	3.5	6.8	11.0	12.5	8.7	**2** **×** **10** ^**−4**^	**4** **×** **10** ^**−3**^	**8** **×** **10** ^**−5**^	**1** **×** **10** ^**−8**^	8 × 10^−1^
Loss of control	7.6	6.4	9.5	13.0	14.2	11.1	**6** **×** **10** ^**−3**^	**2** **×** **10** ^**−2**^	**4** **×** **10** ^**−20**^	**7** **×** **10** ^**−13**^	**1** **×** **10** ^**−9**^
Continued use despite adverse consequences	5.2	3.3	8.2	10.6	11.2	9.7	**1** **×** **10** ^**−7**^	2 **×** 10^−1^	**6** **×** **10** ^**−14**^	**4** **×** **10** ^**−12**^	**2** **×** **10** ^**−5**^
Loss of interest in previous hobbies	1.8	0.9	3.4	1.9	2.2	1.5	**4** **×** **10** ^**−6**^	2 **×** 10^−1^	**1** **×** **10** ^**−6**^	**4** **×** **10** ^**−6**^	**1** **×** **10** ^**−2**^
Use of internet to escape or relieve a negative mood	8.6	7.4	10.6	12.8	15.3	8.8	**6** **×** **10** ^**−3**^	**3** **×** **10** ^**−6**^	**1** **×** **10** ^**−22**^	**2** **×** **10** ^**−23**^	**1** **×** **10** ^**−4**^
Dissimulation	1.8	1.0	3.2	1.5	1.7	1.2	**7** **×** **10** ^**−5**^	3 **×** 10^−1^	**3** **×** **10** ^**−2**^	**2** **×** **10** ^**−3**^	1
Jeopardy or loss of a significant relationship	1.1	0.5	2.1	1.1	1.3	0.9	**3** **×** **10** ^**−4**^	3 **×** 10^−1^	**4** **×** **10** ^**−2**^	**4** **×** **10** ^**−3**^	1

The *p* values show the results from the *χ* ^2^ or Fisher’s exact tests. *p* < 5 **×** 10^−2^ in bold print

*WS* whole sample

As expected, there were strong positive Spearman’s correlations between the number of affirmed IGD and SND criteria and the time spent on internet gaming or social networks, the CIUS score and age for both sexes (Table 3).

**Table 3:** Spearman’s correlations of the affirmed IGD and SND criteria with internet use times and CIUS score

Number of affirmed criteria	Times spent on the respective internet activity				CIUS		Age	
	Average		Maximum					
	♀	♂	♀	♂	♀	♂	♀	♂
IGD	*ρ* = .602	*ρ* = .658	*ρ* = .614	*ρ* = .632	*ρ* = .288	*ρ* = .373	*ρ* = −.071	*ρ* = −.311
	*p* < 10^−99^	*p* < 10^−99^	*p* < 10^−99^	*p* < 10^−99^	*p* = 2 × 10^−30^	*p* = 2 × 10^−32^	*p* = 6 × 10^−3^	*p* = 1 × 10^−22^
SND	*ρ* = .468	*ρ* = .489	*ρ* = .485	*ρ* = .486	*ρ* = .461	*ρ* = .327	*ρ* = −.302	*ρ* = −.248
	*p* = 4 × 10^−82^	*p* = 3 × 10^−56^	*p* = 1 × 10^−89^	*p* = 4 × 10^−56^	*p* = 4 × 10^−81^	*p* = 7 × 10^−25^	*p* = 1 × 10^−33^	*p* = 1 × 10^−14^

These findings were confirmed by multivariate analyses including potential confounders. The numbers of affirmed IGD and SND criteria were associated with the CIUS score and the time spent on internet gaming (MANCOVA, *n* = 2,253: *F* = 505, *p* < 10^−99^, Wilk’s *Λ* = .595, partial *η* ^2^ = .405) and social networks (*n* = 2,227: *F* = 231, *p* < 10^−99^, Wilk’s *Λ* = .761, partial *η* ^2^ = .239). More specifically, the number of IGD criteria related to the CIUS score (*F* = 177, *p* = 7 x 10^−39^, partial *η* ^2^ = .074) and the average (*F* = 1,290, *p* < 10^−99^, partial *η* ^2^ = .367) and maximum (*F* = 1,276, *p* < 10^−99^, partial *η* ^2^ = .367) time spent on internet games. The male participants reported significantly longer maximum time spent on internet games compared with the females. Moreover, the proposed SND criteria related to the CIUS score (*F* = 277, *p* = 1 × 10^−58^, partial *η* ^2^ = .112) and the average (*F* = 418, *p* = 4 × 10^−85^, partial *η* ^2^ = .159) and maximum (*F* = 427, *p* = 8 × 10^−87^, partial *η* ^2^ = .162) time spent on social networks. Here, we found significantly lower CIUS scores in females than in males and in ex-smoker than in smoker participants.

**Fig. 1:**
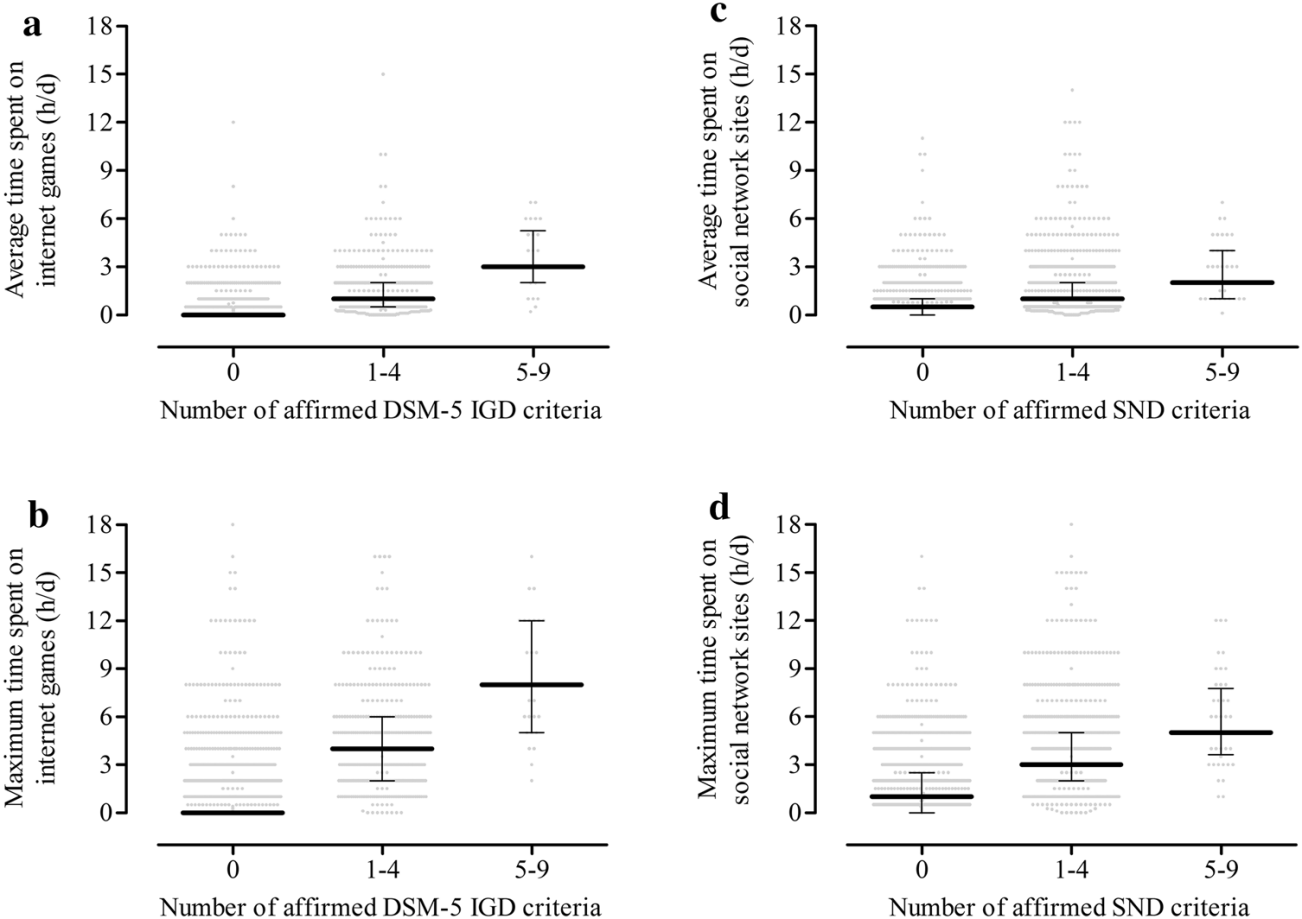
Relationship between the DSM-5 and the adapted criteria for IGD and SND and the time invested on internet gaming or social networks The subgroups based on the number of affirmed IGD or SND criteria differed in the time spent on internet games [**a**, **b** average, Kruskal–Wallis test: *χ* ^2^ = 972, *p* < 10^−99^; maximum, Kruskal–Wallis test: *χ* ^2^ = 963, *p* < 10^−99^; significant post hoc results (Dunn’s tests) for “0” vs. “1–4” and “0” vs. “5–9”] and social networks [**c**, **d** average, Kruskal–Wallis test: *χ* ^2^ = 525, *p* < 10^−99^; maximum, Kruskal–Wallis test: *χ* ^2^ = 559, *p* < 10^−99^; significant post hoc results (Dunn’s tests) for “0” vs. “1–4”, “0” vs. “5–9” and “1–4” vs. “5–9”]. The graphs show the medians and the 25/75 percentiles

We then subdivided the participants into groups depending on the number of affirmed IGD and SND criteria (“0”, “1–4”, “5–9”) and found significant differences (Fig. 1). The participants who affirmed 5–9 criteria reported that they spent a median maximum time on internet games of 8.0 h/day (25/75 % percentiles 5.0/12.0) and a median maximum time on social networks of 5.0 h/day (3.6/7.8).

Altogether, 1.1 % [(10♀ + 17♂)/2,465] of the participants suffered from IGD, and 1.8 % [(35♀ + 10♂)/2,465] suffered from SND. In comparison with the females, the males showed an increased prevalence of IGD (♂ 1.8 vs. ♀ 0.7 %, *p* = 8 × 10^−3^) and a decreased prevalence of SND (♂ 1.1 vs. ♀ 2.3 %, *p* = 3 × 10^−2^). The Youden’s index for IGD was maximal at 3.75 h/day (area under the curve [AUC] .943; sensitivity .926; specificity 0.844, *p* = 2 × 10^−15^) and for SND at 2.75 h/day (AUC .830; sensitivity .909; specificity 0.612, *p* = 6 × 10^−14^). Overall, 61.4 % [(1,012♀ + 501♂)/2,465] of the participants denied the use of internet games during the preceding year, and 17.4 % [(241♀ + 189♂)/2,465] denied the use of social networks.

### IGD, SND and health status

We were interested in the impact of pathological internet use on an individual’s life and used two thresholds for a classification of pathological: (1) the number of affirmed criteria (“0–4” vs. “5–9”) and (2) the time spent on the internet (“<30” vs. “≥30 h/week”) based on our ROC curves [sensitivity/specificity, IGD_(31.5 h/week)_ .852/.884, SND_(29.75 h/week)_ .636/.843) and the cutoffs suggested by Rumpf et al. (2013). The analyses revealed that the participants who affirmed ≥5 of the IGD or SND criteria suffered significantly more often from depression, eating disorders and burnout. Spending ≥ 30 h/week on social networks related to increased risks for depression, obsessive–compulsive disorders and eating disorders (Table 4). Sex-specific analyses revealed additional significant associations. The females who affirmed ≥5 criteria were at increased risks of depression [odds ratio (OR) IGD 4.9, *p* = 2 × 10^−2^; SND 3.2, *p* = 5 × 10^−3^], eating disorders (OR SND 5.8, *p* = 2 × 10^−2^), and burnout (OR SND 4.0, 1 × 10^−2^); social network use for ≥30 h/week related to increased risks for depression (OR 1.8, *p* = 2 × 10^−3^), eating disorders (OR 2.5, *p* = 4 × 10^−2^) and other psychiatric disorders (OR 2.4, *p* = 6 × 10^−4^). The males who affirmed ≥5 IGD criteria were at increased risks of depression (OR 4.8, *p* = 1 × 10^−2^) and panic/anxiety disorder (OR 4.5, *p* = 4 × 10^−2^).

**Table 4:** Comparison of the frequency of psychiatric disorders associated with IGD and SND

	IGD							SND							
	Criteria 5-9				Time ≥ 30 h/week			Criteria 5–9				Time ≥ 30 h/week			
	%	OR	*p*		%	OR	*p*	%	OR	p		%	OR	*p*	
Depression	33.3	4.3	**1** × **10** ^**−3**^	♀♂	9.9	0.9	7 × 10^−1^	27.3	3.2	**2** × **10** ^**−3**^	♀	14.3	1.5	**1** × **10** ^**−2**^	♀
Mania	0	–	1		0.3	1.2	1	0	–	1		0.3	0.8	1	
Psychosis/schizophrenia	0	–	1		0.3	1.4	6 × 10^−1^	0	–	1		0.5	2.5	3 × 10^−1^	
Substance use disorder	0	–	1		0.3	0.4	7 × 10^−1^	0	–	1		1.0	1.4	5 × 10^−1^	
Panic/anxiety disorder	11.1	2.1	2 × 10^−1^	♂	7.0	1.3	3 × 10^−1^	6.8	1.2	7 × 10^−1^		6.0	1.1	7 × 10^−1^	
OCD	3.7	9.2	1 × 10^−1^		0.7	1.6	6 × 10^−1^	2.3	5.5	2 × 10^−1^		1.3	4.2	**2** × **10** ^**−2**^	
Eating disorder	0	–	1		0.7	0.5	6 × 10^−1^	6.8	6.9	**1** × **10** ^**−2**^	♀	2.5	2.8	**2** × **10** ^**−2**^	♀
Burnout	7.4	2.0	3 × 10^−1^		2.6	0.7	3 × 10^−1^	13.6	4.2	**6** × **10** ^**−3**^	♀	4.5	1.2	4 × 10^−1^	
ADHD	0	–	1		0.7	1.0	1	2.3	3.7	3 × 10^−1^		0.8	1.2	7 × 10^−1^	
Other	7.4	1.8	3 × 10^−1^		2.6	0.6	1 × 10^−1^	9.1	2.3	1 × 10^−1^		6.5	1.7	**2** × **10** ^**−2**^	♀

Table 3 shows the prevalence and odds ratios (OR) of psychiatric disorders in the participants with pathological internet use. “♀” and “♂” denote significant results in the sex-specific analyses (see text for details). The *p*-values illustrate the results from the *χ*^2^ or Fisher’s exact tests. *p* < 5 × 10^−2^ in bold print. Missing data < 2.7 %

*OCD* obsessive–compulsive disorder, *ADHD* attention deficit hyperactivity disorder

Except that the participants who affirmed ≥5 of the criteria for SND affirmed more of the CAGE criteria for a misuse of alcohol (*p* = 3 × 10^−3^), there were no further significant differences between the groups regarding CAGE scores or smoking status. With regard to the use of mental health services, a higher frequency of medical and psychological consultations during the preceding year for mental disorders was related to ≥5 affirmed criteria for IGD (*p* = 3 × 10^−2^) and SND (*p* = 3 × 10^−4^). The participants who used social networks ≥30 h/week reported searching for professional help for mental disorders more frequently (*p* = 2 × 10^−3^). Moreover, there were no group differences in terms of the self-estimated health status, the number of doctors’ visits due to somatic morbidities during the previous 12 months or the time spent on sport or sleep. Finally, we found that belonging to the risk group with ≥30 h/week spent on internet games was associated with less working hours/week (*p* = 4 × 10^−3^) and that spending ≥30 h/week on social networks was associated with less working hours/week (*p* = 3 × 10^−2^) and less months of employment during the preceding year (*p* = 1 × 10^−3^).

### Laterality and internet use

In a first step, we explored the Spearman’s correlations between the markers of laterality and internet use in a discovery sample of 532 females and 258 males, with complete data on all of the details [median (25/75 % percentiles): age 29 years (24/41); BMI 23 kg/m^2^ (21/26)]. Left-handedness was associated with a longer maximum time spent on social networks (*ρ* = −.074, *p* = 4 × 10^−2^), and a preference for right-side kissing was associated with higher CIUS scores (*ρ* = .078, *p* = 3 × 10^−2^). Stronger left-handed females affirmed more SND criteria (*ρ* = −.087, *p* < 5 × 10^−2^) and their stronger ocular right lateralization (Miles test) was associated with higher CIUS scores (*ρ* = .091, *p* = 4 × 10^−2^). In males, left-handedness related to a longer average (*ρ* = −.150, *p* = 2 × 10^−2^) and maximum time spent on social networks (*ρ* = −.194, *p* = 2 × 10^−3^), and left-footedness related to a longer maximum time spent on internet games (*ρ* = −.123, *p* < 5 × 10^−2^). The multivariate model confirmed that—among all of the laterality markers—primary handedness related to the SND criteria (*F* = 8, *p* = 6 × 10^−3^, partial *η* ^2^ = .010) and maximum time (*F* = 6, *p* = 1 × 10^−2^, partial *η* ^2^ = .008) spent on social networks (MANCOVA: *F* = 2, *p* = 4 × 10^−2^, Wilk’s *Λ* = .981, partial *η* ^2^ = .019). Footedness was also associated with the number of affirmed SND criteria (*F* = 5, *p* = 3 × 10^−2^, partial *η* ^2^ = .006). No other laterality marker showed any significant influence on the CIUS score, internet gaming or the use of social networks. The model was strongly influenced by age (*F* = 40, *p* = 8 × 10^−48^, Wilk’s *Λ* = .730, partial *η* ^2^ = .270), sex (*F* = 2, *p* = 2 × 10^−2^, Wilk’s *Λ* = .978, partial *η* ^2^ = .022) and the CAGE score (*F* = 7, *p* = 2 × 10^−7^, Wilk’s *Λ* = .943, partial *η* ^2^ = .057).

Using an enlarged sample of 1,439 females and 891 males [median (25/75 % percentiles): age 28 years (23/41); BMI 23 kg/m^2^ (21/26)], we found a similar association of handedness with the SND criteria (*F* = 5, *p* = 3 × 10^−2^, partial *η* ^2^ = .002) and the average (*F* = 6, *p* = 1 × 10^−2^, partial *η* ^2^ = .003) and maximum (*F* = 12, *p* = 5 × 10^−4^, partial *η* ^2^ = .005) time spent on social networks (MANCOVA, *F* = 4, *p* = 5 × 10^−3^, Wilk’s *Λ* = .994, partial *η* ^2^ = .006). As expected, this model was also strongly influenced by age (*F* = 202, *p* < 10^−99^, Wilk’s *Λ* = .793, partial *η* ^2^ = .207), CAGE score (*F* = 13, *p* = 4 × 10^−8^, Wilk’s *Λ* = .984, partial *η* ^2^ = .016) and sex (*F* = 9, *p* = 9 × 10^−6^, Wilk’s *Λ* = .989, partial *η* ^2^ = .011). To avoid bias by psychiatric morbidities, we repeated this analysis after exclusion of participants who reported to be affected and found a similar link between handedness and use of social networks [*n* (♀) = 1,133, *n* (♂) = 754; MANCOVA, *F* = 3, *p* = 2 × 10^−2^, Wilk’s *Λ* = .995, partial *η* ^2^ = .005].

**Fig. 2:**
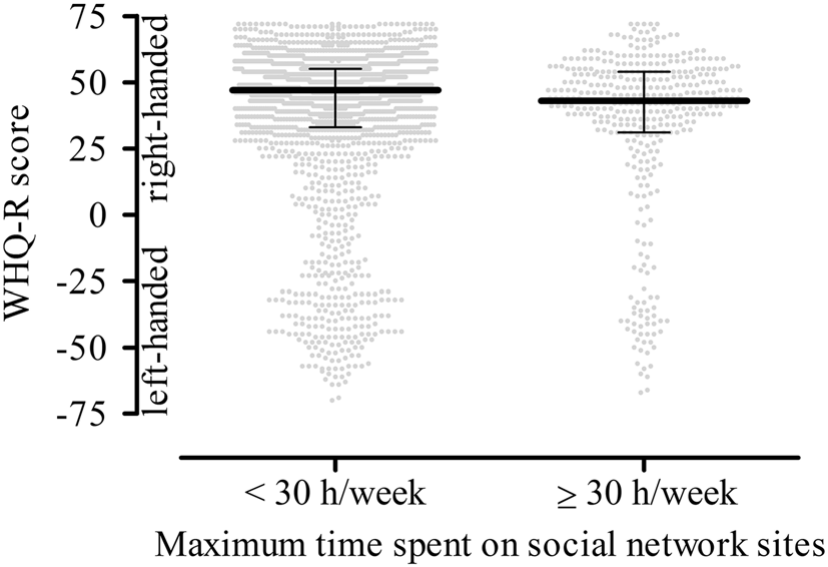
Difference in handedness between the healthy and excessive users of social networks. The participants who reported spending ≥30 h/week (h/week) on social networks scored lower on the waterloo handedness questionnaire-revised (WHQ-R) and were thus more strongly left-handed than participants who stated using social networks <30 h/week (Mann–Whitney *U* test, *U* = 337,810, *p* = 3 × 10^−3^). The graph shows the medians and the 25/75 percentiles

Finally, the participants who reported a maximum time spent on social networks ≥30 h/week during the previous 12 months were more strongly left-handed compared with the never or rare social network users (ANCOVA: *F* = 6, *p* = 1 × 10^−2^, partial *η* ^2^ = .003; Fig. 2).

## Discussion

### Applicability and characterization of the DSM-5 criteria for IGD and the educed SND criteria

With the inclusion of IGD in the DSM-5, the APA encouraged empirical research on the proposed IGD criteria (American Psychiatric Association 2013). The neglect of other internet activities such as chatting leads to a one-sided diagnostic approach that has not been approved scientifically (Thomasius et al. 2014). Therefore, our first aim was to examine the applicability of the DSM-5 research criteria for IGD and the adapted criteria for SND. Overall, the provided criteria proved to be user-friendly and comprehensible in our study. The internal consistency reliability (KR-20 coefficient) was .716 for the DSM-5 IGD criteria and .666 for the SND criteria. The core components of addiction such as “salience, mood modification, tolerance, withdrawal, conflict and relapse” (Griffiths 2005) entail increased times of use. Accordingly, the pathological users in our study reported significantly longer times spent on the internet than did the healthy users. The strong correlations of the IGD and the SND criteria with the CIUS scores and the time spent on the respective internet activities corroborate the concept of internet use disorders. Moreover, the subgroups based on the number of affirmed criteria (“0”, “1–4”, “5–9”) differed significantly. The cut-off value of 5/9 criteria proved to be useful because the “5–9” SND group in particular reported a significantly higher time spent on social networks compared with the “0” and “1–4” groups. The prevalence of 1.1 % for IGD and 1.8 % for SND is in line with other independent conservative reports of Western countries (Aboujaoude et al. 2006; Bakken et al. 2009; Rumpf et al. 2013; Sussman et al. 2011). As expected, age and sex showed strong effects. In line with previous reports (Andreassen et al. 2012, 2013), younger age related to more affirmed IGD and SND criteria. The male participants were more likely to affirm several IGD criteria and report more time spent on internet games; the female participants affirmed SND criteria more often (Table 2). These findings agree with other studies showing that males are at greater risk to develop IGD (Batthyàny et al. 2009; Wenzel et al. 2009) and that females are jeopardized more often by SND (Durkee et al.  2012). Whether the use of the internet is pathological depends on the consequences that it carries for life. Hence, it is important to mention that the participants who were classified as pathological users (5–9 IGD, 5–9 SND criteria or ≥30 h/week spent on the respective internet activity) were significantly more likely to consult professionals for depression, obsessive–compulsive disorder, eating disorder, burnout and panic/anxiety disorder. In line with previous findings on comorbid psychiatric disorders in excessive internet users (Carli et al. 2013; Morrison and Gore 2010; Spada 2014; Weinstein and Lejoyeux 2010), our report notes that pathological internet use negatively impacts mental health. Finally, our observation that ≥30 h/week spent on internet activities relates to significantly fewer working hours/week and to fewer months of employment during the preceding year further illustrates how pathological internet use may impair an individual’s life.

### Laterality and internet use

The second goal of this study was to explore whether internet use in general, internet gaming and/or the use of social networks relate to the variables of cerebral lateralization. We investigated a broad range of markers (handedness, footedness, eyedness, earedness, rotational preference in gymnastics and head-turning asymmetry) and were mindful of potential confounding factors such as sex, age, smoking and drinking status, educational status and body mass index. Within a discovery sample (*n* = 790), we detected significant associations of handedness with the number of affirmed SND criteria and the time spent on social networks. This association was also found in the enlarged sample of 2,330 participants, but we acknowledge the small effect sizes (Spearman’s *ρ* = −.194 to −.074; MANCOVA *η* ^2^ = .002–.010). Moreover, those who spent ≥30 h/week on social networks were significantly more strongly left-handed. As far as we know, this is the first investigation that identifies left-handedness as a risk factor for pathological use of social networks. Although it remains to be specified whether handedness and prenatal testosterone correlate positively or negatively (Geschwind and Galaburda 1985; Witelson 1985), the results of this study agree with our early sex hormone model of addiction. Accordingly, a recent study reported pathological social network use to relate to impulsivity (Wu et al. 2013), which is a strongly sex-specific behavioral trait that is influenced by prenatal testosterone exposure (Wacker et al. 2013) and is linked to substance-related addictions (Lejuez et al. 2010; Potenza and de Wit 2010). Nevertheless, we found no significant association between laterality markers and internet gaming. This may be due to the relatively small number of males within the discovery sample (*n* = 258). Another reason might be that social reinforcement, which is a main factor regarding the internet’s addictive potential (Morahan-Martin and Schumacher 2000), is more relevant to social networks than to internet games.

### Strengths and limitations

The large cohort enabled us to control for several potentially influencing demographic variables. The anonymity of the participants reduced confounding by social stigmatization. However, the sample is not representative of the general population, and attention is advised against over-generalization. The recruitment partly through e-mail, internet ads, social networks and the online survey entails a selection toward internet users. The investigated cohort is also characterized by a high educational level. Moreover, all of the implemented measures were self-report questionnaires, and we cannot rule out careless or wrong answers despite the application of several quality measurements. Finally, the cross-sectional study design does not allow us to estimate the test–retest reliability of the criteria or draw conclusions about causalities that underlie the development of pathological internet use.

In summary, we successfully applied and characterized the DSM-5 research criteria for IGD and the adapted criteria for SND. Their associations with comorbid psychiatric disorders demand stronger attention to pathological use of internet games and social networks in clinical practice. Moreover, we found that the use of social networks is related to handedness, which is in line with our early sex hormone model of addiction and supports the classification of pathological internet use as an addictive disorder.

## Supplementary Information

Below is the link to the electronic supplementary material.

Supplementary file1 (DOC 178 KB)
