# Supplementary material for: Internet gaming disorder, social network disorder and laterality: handedness relates to pathological use of social networks
Source: J Neural Transm (Vienna). 2015 Jan 10;122(8):1187–96. doi: 10.1007/s00702-014-1361-5 (PMC4513228; doi:10.1007/s00702-014-1361-5)
Supplement: Supplementary file 1 — Supplementary file1 (DOC 178 KB) [file 702_2014_1361_MOESM1_ESM.doc]

German Translation of the Waterloo Handedness Questionnaire-Revised (WFQ-R)

Versuchen Sie, sich selbst bei den folgenden Tätigkeiten vorzustellen oder diese pantomimisch auszuprobieren. Welche **Hand** nutzen Sie bevorzugt bei den folgenden Tätigkeiten? Entscheiden Sie sich bitte zwischen immer („nur“, mindestens 95% der Gelegenheiten), gewöhnlich („häufig“, ca. 75% der Gelegenheiten) und gleich oft („beide“).

|  | nur | häufig |  | häufig | nur |
| --- | --- | --- | --- | --- | --- |
|  | links | links | beide | rechts | rechts |
| Einen Lautstärkeregler am Radio bedienen |  |  |  |  |  |
| Eine Wand mit einem Pinsel anmalen |  |  |  |  |  |
| Eine Suppe löffeln |  |  |  |  |  |
| Mit dem Finger auf etwas in der Ferne zeigen |  |  |  |  |  |
| Einen Dartpfeil werfen |  |  |  |  |  |
| Den Radiergummi am Bleistiftende verwenden |  |  |  |  |  |
| Einen Spazierstock halten |  |  |  |  |  |
| Mit einem Bügeleisen ein Hemd bügeln |  |  |  |  |  |
| Ein Bild zeichnen |  |  |  |  |  |
| Eine Kaffeetasse voller Kaffee halten |  |  |  |  |  |
| Einen Nagel in ein Stück Holz einschlagen |  |  |  |  |  |
| Eine Fernbedienung benutzen |  |  |  |  |  |
| Mit einem Messer Brot schneiden |  |  |  |  |  |
| Buchseiten umblättern |  |  |  |  |  |
| Mit einer Schere Papier schneiden |  |  |  |  |  |
| Eine Tafel sauber wischen |  |  |  |  |  |
| Mit einer Pinzette greifen |  |  |  |  |  |
| Ein Buch in die Hand nehmen |  |  |  |  |  |
| Einen Koffer tragen |  |  |  |  |  |
| Kaffee in eine Tasse einschenken |  |  |  |  |  |
| Eine Computermaus bedienen |  |  |  |  |  |
| Einen Stecker in die Steckdose stecken |  |  |  |  |  |
| Eine Münze werfen |  |  |  |  |  |
| Mit einer Zahnbürste die Zähne bürsten |  |  |  |  |  |
| Einen Baseball werfen |  |  |  |  |  |
| Einen Türknopf drehen |  |  |  |  |  |
| Schreiben |  |  |  |  |  |
| Ein Blatt Papier in die Hand nehmen |  |  |  |  |  |
| Eine Handsäge verwenden |  |  |  |  |  |
| Flüssigkeit mit einem Löffel umrühren |  |  |  |  |  |
| Einen offenen Regenschirm tragen |  |  |  |  |  |
| Eine Nadel beim Nähen halten |  |  |  |  |  |
| Ein Streichholz anzünden |  |  |  |  |  |
| Einen Lichtschalter betätigen |  |  |  |  |  |
| Eine Schublade öffnen |  |  |  |  |  |
| Die Tasten eines Taschenrechners drücken |  |  |  |  |  |

Sollten Sie in Ihrer Kinder- und Jugendzeit Dinge mit der rechten Hand erledigen, obwohl Ihnen die Ausführung mit links leichter gefallen wäre? Oder gibt es irgendeinen Grund, weswegen Sie im Laufe der Zeit für eine der oben genannten Aktivitäten die bevorzugte Hand verändert haben (z.B. Verletzung)?

ja nein

Falls ja, bitte kurz erklären: ___________________________________________________

German Translation of the Waterloo Footedness Questionnaire-Revised (WFQ-R)

Welchen **Fuß** bzw. welches **Bein** nutzen Sie bevorzugt bei den folgenden Tätigkeiten? Entscheiden Sie sich bitte zwischen immer („nur“, mindestens 95% der Gelegenheiten), gewöhnlich („häufig“, ca. 75% der Gelegenheiten) und gleich oft („beide“). Versuchen Sie, sich die Aktivitäten möglichst gut vorzustellen oder pantomimisch auszuprobieren.

|  | nur | häufig |  | häufig | nur |
| --- | --- | --- | --- | --- | --- |
|  | links | links | beide | rechts | rechts |
| Einen ruhenden Ball auf ein Ziel schießen |  |  |  |  |  |
| Auf einem Bein stehen (Einbeinstand) |  |  |  |  |  |
| Sand am Strand glätten |  |  |  |  |  |
| Auf einen Stuhl steigen |  |  |  |  |  |
| Auf ein bodennahes wegfliegendes Papierblatt treten, um es festzuhalten |  |  |  |  |  |
| Auf einer Eisenbahnschiene balancieren |  |  |  |  |  |
| Einen Stein mit den Zehen ergreifen |  |  |  |  |  |
| Auf einem Bein hüpfen |  |  |  |  |  |
| Einen Spaten in den Boden stoßen |  |  |  |  |  |
| Bei entspanntem Stehen verlagern die meisten ihr Gewicht auf einen Fuß und entlasten den anderen. Auf welchem Fuß ist bei Ihnen mehr Gewicht? |  |  |  |  |  |

Wurden Sie besonders ermutigt oder trainiert, um einen speziellen Fuß oder ein spezielles Bein für bestimmte Aktivitäten zu benutzen? Oder gibt es irgendeinen Grund, weswegen Sie im Laufe der Zeit für eine der oben genannten Aktivitäten den bevorzugten Fuß oder das bevorzugte Bein verändert haben (z.B. Verletzung)?

ja nein

Falls ja, bitte kurz erklären: ___________________________________________________

German questions for other markers of laterality

*Miles Test:*

Führen Sie bitte folgenden kurzen Test durch: Strecken Sie beide Arme aus und positionieren Sie die Hände so, dass zwischen den beiden Daumen und Zeigefingern eine Dreiecksöffnung entsteht. Öffnen Sie bewusst beide Augen und fixieren Sie durch dieses Dreieck hindurch einen etwa 3 Meter entfernten Gegenstand (siehe Stern im Bild).

Schließen und öffnen Sie nun abwechselnd das rechte und linke Auge und beobachten Sie, ob sich die Position des Gegenstands verändert. Wiederholen Sie diese Übung mit einem zweiten und dritten Gegenstand im Fokus.

Beim Schließen und Öffnen welches Auges verändert sich die Position des fokussierten Gegenstandes?

|  | nur beim linken Auge | links stärker als rechts | bei beiden Augen gleich | rechts stärker als links | nur beim rechten Auge | keine Angabe |
| --- | --- | --- | --- | --- | --- | --- |
| Gegenstand 1 |  |  |  |  |  |  |
| Gegenstand 2 |  |  |  |  |  |  |
| Gegenstand 3 |  |  |  |  |  |  |


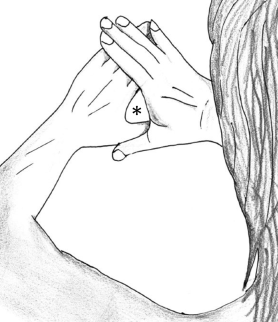


*Kissing side preference:*

Man hat beobachtet, dass Menschen ihren Kopf beim Küssen auf den Mund bevorzugt zu einer Seite neigen. Stellen Sie sich vor, wie Sie jemandem gegenüber stehen und sie/ihn auf den Mund küssen (siehe Bild). Wohin dreht sich dabei Ihr Kopf?

| nur nach links (≥95% der Küsse) | häufig nach links (~75% der Küsse) | etwa gleich häufig nach links oder rechts | häufig nach rechts (~75% der Küsse) | nur nach rechts (≥95% der Küsse) | keine Angabe |
| --- | --- | --- | --- | --- | --- |

*Side preference in sports activities:*

Stellen Sie sich vor, Sie wollen hochspringen und dabei etwa eine halbe Drehung ausführen (siehe Abbildung). In welche Richtung würden Sie sich dabei drehen: nach rechts, d.h. im Uhrzeigersinn oder nach links, also gegen den Uhrzeigersinn?

| nur nach links | häufig nach links | auf beide Seiten | häufig nach rechts | nur nach rechts | Ich kann nicht beidbeinig springen. |
| --- | --- | --- | --- | --- | --- |


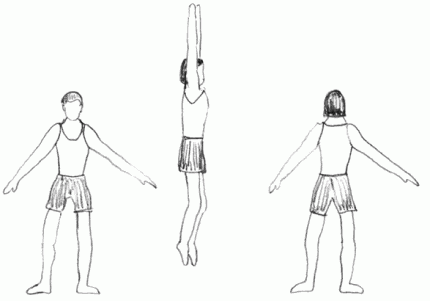

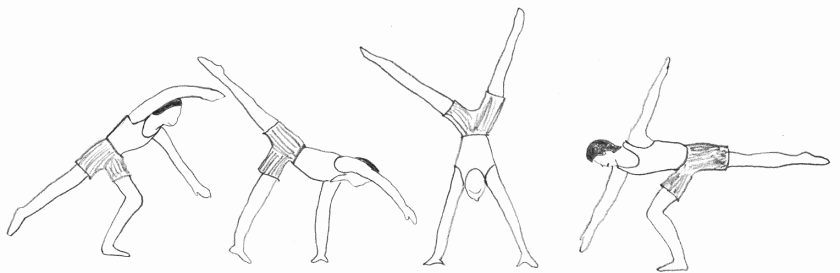


Falls Sie im Turnen ein Rad schlagen können: Berührt dann Ihre rechte oder Ihre linke Hand als erstes den Boden?

| nur die linke | häufig die linke | etwa gleich häufig die linke und rechte | häufig die rechte | nur die rechte | Ich kann kein Rad schlagen. |
| --- | --- | --- | --- | --- | --- |

German questionnaire for Internet Gaming Disorder

Mit den folgenden Fragen wollen wir erfahren, welche Bedeutung Internetspiele (Onlinespiele am Computer) in Ihrem Leben haben. Beziehen Sie sich bitte nur auf die Zeit, in der Sie das Internet in Ihrer Freizeit nutzen und schließen Sie berufliche Nutzung aus. Wenn Sie irgendwann einmal in dem Zeitraum der letzten zwölf Monate der Frage zugestimmt hätten, beantworten Sie diese bitte mit „Ja“.

|  | ja | nein |
| --- | --- | --- |
| Müssen Sie ständig an das Internetspielen denken, auch in Lebensphasen, in denen Sie nicht spielen können (z.B. in der Schule oder am Arbeitsplatz)? |  |  |
| Erleben Sie psychische Entzugssymptome wie Gereiztheit, Unruhe, Traurigkeit, erhöhte Ängstlichkeit oder Konzentrationsprobleme, wenn Sie nicht im Internet spielen können? |  |  |
| Haben Sie im Laufe der Zeit das Bedürfnis verspürt, mehr und mehr Zeit mit dem Internetspielen zu verbringen? |  |  |
| Gelingt es Ihnen nicht, die Häufigkeit und Dauer des Internetspielens zu begrenzen und selbst zu bestimmen, wann Sie mit dem Spielen beginnen und wann Sie damit aufhören? |  |  |
| Spielen Sie weiter im Internet, obwohl Sie wissen, dass es nachteilige psychosoziale Auswirkungen (z.B. auf Ihre Leistungsfähigkeit am Arbeitsplatz oder auf Ihr psychisches Wohlbefinden) für Sie hat? |  |  |
| Haben Sie Ihr Interesse an früher geschätzten Hobbies und Freizeitaktivitäten verloren und interessieren Sie sich nur noch für das Internetspielen? |  |  |
| Spielen Sie im Internet, um negative Gefühle zu steuern und Probleme zu vergessen? |  |  |
| Belügen Sie Familienmitglieder, Therapeuten oder andere Personen über das tatsächliche Ausmaß Ihres Internetspielens? |  |  |
| Haben Sie wegen des Internetspielens wichtige Beziehungen, Karrierechancen oder Ihren Arbeitsplatz riskiert oder verloren oder Ihre Zukunft in anderer Weise gefährdet? |  |  |

German questionnaire for Social Network Disorder

Nun geht es darum, welche Bedeutung Chatten und Mailen in sozialen Netzwerken (elektronische Textkommunikation in Echtzeit z.B. Facebook, Twitter, WhatsApp, Hangout oder andere Messenger-Dienste auf PC, Tablet oder Smartphone) für Ihr Leben haben. Beziehen Sie sich bitte auch hier nur auf die Zeit, in der Sie das Internet in Ihrer Freizeit nutzen und schließen Sie berufliche Nutzung aus. Wenn Sie irgendwann einmal in dem Zeitraum der letzten zwölf Monate der Frage zugestimmt hätten, beantworten Sie diese bitte mit „Ja“.

|  | ja | nein |
| --- | --- | --- |
| Müssen Sie ständig an das Chatten und Mailen denken, auch in Lebensphasen, in denen Sie nicht Chatten und Mailen können (z.B. in der Schule oder am Arbeitsplatz)? |  |  |
| Erleben Sie psychische Entzugssymptome wie Gereiztheit, Unruhe, Traurigkeit, erhöhte Ängstlichkeit oder Konzentrationsprobleme, wenn Sie nicht chatten und mailen können? |  |  |
| Haben Sie im Laufe der Zeit das Bedürfnis verspürt, mehr und mehr Zeit mit Chatten und Mailen zu verbringen? |  |  |
| Gelingt es Ihnen nicht, die Häufigkeit und Dauer des Chattens und Mailens zu begrenzen und selbst zu bestimmen, wann Sie mit dem Chatten und Mailen beginnen und wann Sie damit aufhören? |  |  |
| Chatten und mailen Sie weiter, obwohl Sie wissen, dass es nachteilige psychosoziale Auswirkungen (z.B. auf Ihre Leistungsfähigkeit am Arbeitsplatz oder auf Ihr psychisches Wohlbefinden) für Sie hat? |  |  |
| Haben Sie Ihr Interesse an früher geschätzten Hobbies und Freizeitaktivitäten verloren und interessieren Sie sich nur noch für das Chatten und Mailen? |  |  |
| Chatten und mailen Sie, um negative Gefühle zu steuern und Probleme zu vergessen? |  |  |
| Belügen Sie Familienmitglieder, Therapeuten oder andere Personen über das tatsächliche Ausmaß Ihres Chattens und Mailens? |  |  |
| Haben Sie wegen des Chattens und Mailens wichtige Beziehungen, Karrierechancen oder Ihren Arbeitsplatz riskiert oder verloren oder Ihre Zukunft in anderer Weise gefährdet? |  |  |
